# Adherence to European Association of Urology Guidelines and State of the Art of Glycosaminoglycan Therapy for the Management of Urinary Tract Infections: A Narrative Review and Expert Meeting Report

**DOI:** 10.1016/j.euros.2022.07.009

**Published:** 2022-08-23

**Authors:** Gernot Bonkat, Tommaso Cai, Carlotta Galeone, Bela Koves, Franck Bruyere

**Affiliations:** a Alta uro AG, Merian Iselin Klinik, Centre of Biomechanics & Calorimetry, University of Basel, Basel, Switzerland; b Department of Urology, Santa Chiara Hospital, Trento, Italy; c Bicocca Applied Statistics Center (B-ASC), Università degli Studi di Milano-Bicocca, Milano, Italy; d Biostatistics & Outcome Research, Statinfo, Renate, Italy; e Department of Urology, University of Szeged, Szeged, Hungary; f Department of Urology, University of Tours, Tours, France

**Keywords:** Alternative therapies, European Association of Urology guidelines, Urinary tract infections, Survey, Glycosaminoglycans

## Abstract

**Context:**

Urinary tract infections (UTIs) have a significant impact on patient’s quality of life and society. Antibiotic therapy is the primary approach for the management of UTIs; however, it has major limits in the prevention of recurrent UTIs (rUTIs), also increasing the risk of development of multidrug-resistant micro-organisms.

**Objective:**

The aim of this paper is to discuss the European Association of Urology guidelines for the management of UTIs/rUTIs, the level of adherence to these recommendations, and the available evidence on the use of glycosaminoglycans (GAGs) as a possible alternative treatment to prevent rUTIs.

**Evidence acquisition:**

This narrative review and expert meeting report is based on a literature search concerning the currently available UTI guidelines, the results of a survey administered to 227 urologists, and the opinion of an expert panel in the field of UTIs.

**Evidence synthesis:**

Results obtained from the literature search showed that adherence to guidelines is not optimal. The survey demonstrated that antibiotics remain one of the treatments of UTIs. However, most of the urologists are aware of the problem caused by the resistance to antibiotics and prefer alternative methods for the prophylaxis of UTIs. Considering the alternative methods, the authors concluded that GAG therapy is highly effective in preventing rUTIs.

**Conclusions:**

Adherence to the international guidelines is important to align the clinical practice and avoid the spreading of antibiotic resistance. The survey outlines that the misuse and overuse of antibiotics are major problems; an analysis of clinical evidence confirms that GAG therapy is a valuable therapeutic approach to prevent the recurrence of episodes of UTIs and to limit the onset of antibiotic resistance.

**Patient summary:**

Although antibiotic therapy is primarily used for the management of urinary tract infections (UTIs), misuse and overuse of antibiotics are of concern. Adherence to the international guidelines is important to prevent the spreading of antibiotic resistance. Clinical evidence confirms that the use of glycosaminoglycans is a valuable therapeutic approach to prevent UTI recurrence and limit the onset of antibiotic resistance.

## Take Home Message

Adherence to the international guidelines is important to align the clinical practice and avoid the spreading of antibiotic resistance. The survey outlines that the misuse and overuse of antibiotics are major problems; the analysis of clinical evidence confirms that glycosaminoglycan therapy is a valuable therapeutic approach to prevent the recurrence of the episodes of urinary tract infections and to limit the onset of antibiotic resistance.

## 1 Introduction

Urinary tract infections (UTIs) are bacterial infections of the bladder and associated structures [1]. Clinically, UTIs are classified as uncomplicated or complicated. Uncomplicated UTIs, differentiated into lower (cystitis) and upper (pyelonephritis) UTIs, usually affect healthy individuals without structural or neurological urinary tract abnormalities. The most common risk factors are sexual intercourse, spermicide use, having a new sex partner, having a mother with a history of UTIs, having had UTIs during childhood, and asymptomatic bacteriuria treatment [2]. Women are most affected due to the position of the urethra, favoring easier bacterial colonization. Complicated UTIs are associated with factors such as urinary obstruction, urinary retention caused by neurological disease, immunosuppression, renal failure, renal transplantation, pregnancy, and the presence of foreign bodies such as calculi, catheters, or other drainage devices. Uncomplicated and complicated UTIs are most frequently caused by uropathogenic *Escherichia coli* [3].

UTIs are among the most common infectious diseases, with annual costs estimated to be higher than $1.5 billion in the USA [4]. The incidence of UTIs worldwide is estimated to be up to 250 million cases per year [5]; 40–50% of women develop UTIs in their life, 25% of them suffering from recurrent UTIs (rUTIs), diagnosed when three episodes of UTIs occur in the past 12 mo or two episodes in the past 6 mo. [6]. In young women affected by rUTIs, each acute episode is associated with 6 d of symptoms, 2.4 d of restriction of activity, 1.2 d of sick leave (from work or school), and 0–4 d of bed rest [7]. Common symptoms of rUTIs include urinary frequency, urgency, suprapubic discomfort, and dysuria [1]. Moreover, rUTIs cause a high level of anxiety and stress [8], leading to worsened quality of life (QoL) of patients.

Prophylaxis of UTIs includes a wide variety of approaches: antimicrobial, immunoprophylaxis, behavioral measures (fluid intake, pre- or postcoital urination, and hygiene procedures), estrogenic therapy, D-mannose, lactobacillus, acupuncture, urine acidification, herbal drugs, vaccination, phages, and intravesical instillation of medicaments [9], [10]. Chemoprophylaxis of rUTIs consists in the administration of nitrofurantoin, trimethoprim (or cotrimoxazole), and fosfomycin trometamol as first-line drugs. Other options consist in oral or parenteral immunoprophylaxis, together with the use of cranberry products, specific plant combinations, or probiotics [11]. Antibiotics, immunoprophylaxis [11], [12], [13] and estrogenic therapy [10], [14], [15] are supported by scientific literature. For other approaches, such as approaches based on *Lactobacillus* or cranberry extract, there is no concrete evidence on their real efficacy [16]. The use of endovesical instillations of hyaluronic acid (HA) or a combination of HA and chondroitin sulfate (CS) to prevent rUTIs has provided promising results; yet, the level of evidence is still weak and further studies are needed to confirm the results of initial trials. The quality of evidence is higher for the combination than for HA alone [16], [17].

Antimicrobials are the most common treatment of uncomplicated UTIs. Trimethoprim/sulfamethoxazole, trimethoprim, β-lactams, fluoroquinolones, nitrofurantoin, and fosfomycin tromethamine are widely used worldwide [18], [19]. Antibiotics can eradicate bacteria and resolve the problem of UTIs, but while these induce the spread of resistance, these also cannot always prevent recurrences. As widely known, antibiotic resistance is one of the major challenges for public health [20]. It is expected that by 2050, antibiotic resistance will cause up to 10 million deaths per year globally [21], including 60 000 newborns in India only [22]. To understand the extent of the problem of antibiotic resistance, it might be sufficient to think that, according to some estimates, the costs caused by antibiotic resistance are equal to those caused by an increase of 2 °C in global temperature [23]. At the same time, the number of new antibacterial drugs approved is decreasing, leading to the decline of the “golden era” of the antimicrobial therapy. In addition to the above limits, antibiotic treatment can lead to long-term impairment of the normal microbiota of the vagina and gastrointestinal tract [24]. The lack of a standard approach for the management of patients with UTIs/rUTIs and the need to reduce the use of antibiotics suggest rethinking about the current practice in UTI management. A custom approach according to the bacterial characteristics and patient-related risk factors is a promising option for the administration of the best antimicrobial/alternative therapy [25].

## 2 Evidence acquisition

In this narrative review and expert meeting report, the authors first performed a literature search on the currently available guidelines for UTI management, then analyzed the results of a European survey among urologists, and finally discussed the evidence during a symposium at the occasion of the European Association of Urology (EAU) Congress 2021. For the literature search, the keywords used were “UTIs/rUTIs” and “guidelines”; clinical trials, cohort studies, and systematic reviews were excluded. The literature databases consulted were PubMed and Cochrane Library; the results of the past 5 yr were considered. Concerning the survey, the authors reported the results of a structured questionnaire administered to 227 urologists during the EAU/European Lower Urinary Tract Symptoms (ELUTS)/International Society for the Study of Bladder Pain Syndrome (ESSIC) meetings in 2018 to assess real-world prescribing patterns and their opinions on antibiotic resistance. Descriptive statistics was used to analyze the results, providing the percentage of the answers to each question. Finally, the authors, constituting an expert panel, discussed the evidence in the satellite symposium *Are recurrent urinary tract infections a problem in urology? Facts and fiction* held on July 10, 2021, during the virtual congress of the EAU.

## 3 Evidence synthesis

### 3.1 What do EAU guidelines say for the management of rUTIs?

The literature search showed that there are several guidelines available providing recommendations for the management of UTIs. Despite some differences, all guidelines recommend the use of antimicrobial treatments for the acute phase of UTIs, and lifestyle advice, antimicrobial prophylaxis, and nonantimicrobial treatment for the prophylaxis of this infectious disease. With over 16 000 members, EAU is the leading European scientific society on urological practice. The EAU guidelines are endorsed by >70 national societies and scientific organizations including the 27 EU Member States. The EAU guidelines are frequently searched on the Internet, registering >355 000 visits in 6 mo.

The guidelines of the EAU are based on three pivotal concepts for a correct use of antimicrobial prophylaxis: (1) knowledge of the local pathogen profile and antimicrobial resistance, (2) careful evaluation of patient-related risk factors for the development of infectious complications after urological procedures, and (3) adherence to the EAU guidelines on urological infections. The guidelines provide a detailed and updated table summarizing the first-line treatments and alternatives together with the daily dose and the duration of the therapy for UTIs (Table 1) [16].

**Table 1:** First-line treatments and alternatives together with the daily dose and the duration of the therapy for UTIs recommended by the EAU guidelines [16]

Antimicrobial	Daily dose	Duration of the therapy (d)
*First line*		
Fosfomycin trometamol	3 g SD	1
Nitrofurantoin macrocrystal	50–100 mg q.i.d.	5
Nitrofurantoin monohydrate/macrocrystal	100 mg b.i.d.	5
Nitrofurantoin microcrystal ER	100 mg b.i.d.	5
Pivmecillinam	200 mg t.i.d.	3–5
*Alternatives*		
Cephalosporins (eg, cefadroxil)	500 mg b.i.d.	3
*If the local resistance pattern for E. coli is <20%*		
Trimethoprim	200 mg b.i.d.	5
Trimethoprim-sulfamethoxazole	160–180 mg b.i.d.	3

b.i.d. = bis in die (twice a day); EAU = European Association of Urology; q.i.d. = quarter in die (four times a day); SD = single dose; UTI = urinary tract infection.

The guidelines also considered the role of fluoroquinolones, taking into account the characteristics of this class of antibiotics [26] and the increasing concerns about their safety [27], [28]. Fluoroquinolones are broad-spectrum antibiotics and can be used for both Gram-positive and Gram-negative bacteria [26]. These are widely used in urology and for acute uncomplicated cystitis, but despite their popularity, there is an increasing concern regarding the potential severe side effects associated with this class of antibiotics [27]. On October 5, 2018, the European Medicines Agency Pharmacovigilance Risk Assessment Committee recommended restriction of the use of these antibiotics due to the possibility of persistent adverse effects. Over the past decade, the US Food and Drug Administration (FDA) also raised a series of warnings to underline the serious and disabling adverse events associated with fluoroquinolone use. Moreover, in 2018, the FDA required modifications to the labeling of all systemic fluoroquinolones to reinforce warnings about the risk of severe hypoglycemia and mental health effects associated with their use. On March 11, 2019, the European Commission made the regulatory conditions about the use of fluoroquinolones more stringent due to their disabling and potentially long-lasting side effects [28]. The EAU guidelines strongly recommend against the use of aminopenicillins or fluoroquinolones to treat uncomplicated cystitis. However, in uncomplicated cystitis, a fluoroquinolone can be used when the use of other antibacterial agents that are commonly recommended for the treatment of these infections is considered inappropriate [16]. The perioperative antimicrobial prophylaxis according to the EAU guidelines reduces antimicrobial usage without increasing postoperative infection rates, lowers the prevalence of resistant uropathogens, and is cost effective [29]. Unfortunately, the adherence to the EAU guidelines is not optimal. Antimicrobials are often used without a sound rationale, and evidence suggests that >50% of clinicians do not follow the guidelines for the use of antimicrobial agents [30], [31], [32]. The literature search outlined that there are several differences between EAU guidelines and American Urological Association/Canadian Urological Association/Society of Urodynamics, Female Pelvic Medicine & Urogenital Reconstruction guidelines, leading to contradictory and inconsistent recommendations [33].

### 3.2 Assessing real-world prescribing patterns and opinions on antibiotic resistance: results from a European-wide survey

A survey about the management of rUTIs in the clinical practice was conducted on 277 urologists during EAU/ELUTS/ESSIC meetings in 2018. Data were collected using a structured questionnaire consisting of 11 questions (Table 2). The analysis of the survey respondents showed that 76.7% were European and 55.9% were <46 yr old. The most frequent countries of origin are shown in Table 3 (for the complete list of nationalities, see Supplementary Table 1). Most clinicians (55.9%) reported to visit from 20–50 patients affected by lower UTIs per month and that up to 40% of these patients were suffering from rUTIs. The antibiotics remain the cornerstone for the treatment of UTIs, although in clinical practice, they are often used on an empirical basis pending urine culture [34], [35], [36]. In particular, 40.5% of participants reported waiting for an antibiogram test and 43.6% reported starting the antibiotic treatment as soon as possible. International literature frequently reports an inappropriate use of fluoroquinolones and quinolones for UTIs [35], [37]. The survey showed that fluoroquinolones and quinolones are still used by clinicians (these were included in the reply “other” of question 4, corresponding to 51.5%). Of the clinicians, 66.5% reported that they had to change the antibiotic therapy at least “sometimes”. Noteworthy, participants are aware of the problem represented by the resistance to antibiotics (40% of them defined it “extremely relevant”). Of the respondents, 68% noted a slight increase in the trend of antibiotic resistance, while 20% defined it “significantly increasing.” Yet, 60% of clinicians reported to prescribe antibiotics for the prophylaxis of rUTIs. Most of respondents (72.7%) reported to prescribe nonantibiotic methods for the prophylaxis of rUTIs. Details and percentages of the different alternative methods used are reported in Table 2. Urologists above and below 45 yr of age gave similar answers to the questionnaire.

**Table 2:** The management of rUTIs in clinical practice: answers to a survey

Number	Question	Options	Frequency (%)
1	How many patients with an episode of lower UTI do you see in your clinical practice in 1 mo?	<20 20–50 50–100 ˃100	16.3 55.9 19.4 8.4
2	In your opinion, out of 100 patients affected by UTI, how many patients present an rUTI in your clinical practice?	0–20 21–40 41–60 61–80 81–100 No answer	35.7 39.6 12.8 7.9 0.9 3.1
3	When do you usually start antibiotic therapy for the treatment of an rUTI episode?	Always after the antibiogram test As soon as possible, without waiting for antibiogram/culture results Other No answer	40.5 43.6 13.7 2.2
4	Which of the following antibiotic therapies do you usually prescribe for the management of an rUTI episode?	Amoxicillin/clavulanic acid Ampicillin Cotrimoxazole Fosfomycin Nitrofurantoin Pivmecillinam Trimethoprim alone or combined with sulfonamides Other No answer	7.5 0.4 4.0 16.7 11.9 3.1 3.5 51.5 1.3
5	How often have you had to change antibiotic therapy during the treatment of an rUTI episode?	Always Often Sometimes Rarely Never/almost never Not answer	1.8 17.6 66.5 13.7 0 0
6	Do you consider antibiotic resistance a relevant problem in your clinical practice?	Yes, extremely relevant Yes, quite relevant Yes, moderately relevant No, not relevant at all No answer	40.1 36.6 20.3 1.8
7	In your opinion, what has been the trend of antibiotic resistance in your clinical practice in the past 10 yr?	Significantly increasing Slightly increasing Basically unchanged Slightly decreasing Significantly decreasing No answer	18.9 68.3 4.8 2.2 3.5 2.2
8	Do you prescribe antibiotics for the prophylaxis of rUTIs in your clinical practice?	No Yes No answer	38.3 60.3 1.3
9	Do you adopt nonantibiotic methods for the prophylaxis of rUTIs in your clinical practice?	No Yes No answer	23.8 72.7 3.5
10	Based on your experience, how do you rate the efficacy of these nonantibiotic treatments in rUTI management?		
	(a) Endovesical instillation of a combination of hyaluronic acid and chondroitin sulfate	No answer Never prescribed Not effective Moderately effective Extremely effective	29.5 28.2 3.5 25.1 13.7
	(b) Endovesical instillation of chondroitin sulfate	No answer Never prescribed Not effective Moderately effective Extremely effective	38.8 28.2 6.2 21.1 5.7
	(c) Endovesical instillation of hyaluronic acid	No answer Never prescribed Not effective Moderately effective Extremely effective	30.8 20.7 6.2 27.8 14.5
	(d) Prophylaxis with D-mannose	No answer Never prescribed Not effective Moderately effective Extremely effective	35.7 20.7 6.2 29.1 8.4
	(e) Prophylaxis with cranberry	No answer Never prescribed Not effective Moderately effective Extremely effective	23.3 6.2 14.5 44.9 11.0
	(f) Prophylaxis with probiotics (*Lactobacillus* spp.)	No answer Never prescribed Not effective Moderately effective Extremely effective	27.3 8.4 10.6 39.6 14.1
	(g) Immunoactive prophylaxis	No answer Never prescribed Not effective Moderately effective Extremely effective	32.6 18.5 9.7 33.5 5.7
	(h) Hormonal replacement	No answer Never prescribed Not effective Moderately effective Extremely effective	26.0 14.1 6.6 47.6 5.7
11	Do you think that you will continue to prescribe, or you will start to prescribe, nonantibiotic treatment for rUTI management in the future?	Yes, I probably will Perhaps Certainly not I don't know No answer	71.4 21.1 0 3.5 4.0

rUTI = recurrent UTI; UTI = urinary tract infection.

**Table 3:** Most frequent countries of origin of the respondents to the survey

Country	Percentage of respondents
Italy	26.4
Egypt	5.1
UK	4.6
Denmark	3.7
Algeria	3.7
Bulgaria	3.2
Spain	3.2
Slovenia	3.2
Serbia	2.3
Lithuania	2.3

### 3.3 Glycosaminoglycan therapy: a real alternative to antibiotics for the prevention of rUTIs

The satellite symposium *Are recurrent urinary tract infections a problem in urology? Facts and fiction* held on July 10, 2021, during the virtual congress of the EAU, discussed the role of glycosaminoglycan (GAG) therapy as an alternative to antibiotics for the prevention of rUTIs.

In the 1980s, antibiotics protected us from different types of infections. Today, antibiotic resistance fosters the spread of infectious diseases. To overcome this issue, there are two possible approaches. The first one is finding new antibacterial drugs, and yet very few of such medications are being approved. Alternatively, we could change our point of view. Indeed, medicine is changing as life expectancy is increasing; patients are changing too, becoming older and suffering from more comorbidities, and bacteria are changing as well.

**Fig. 1:**
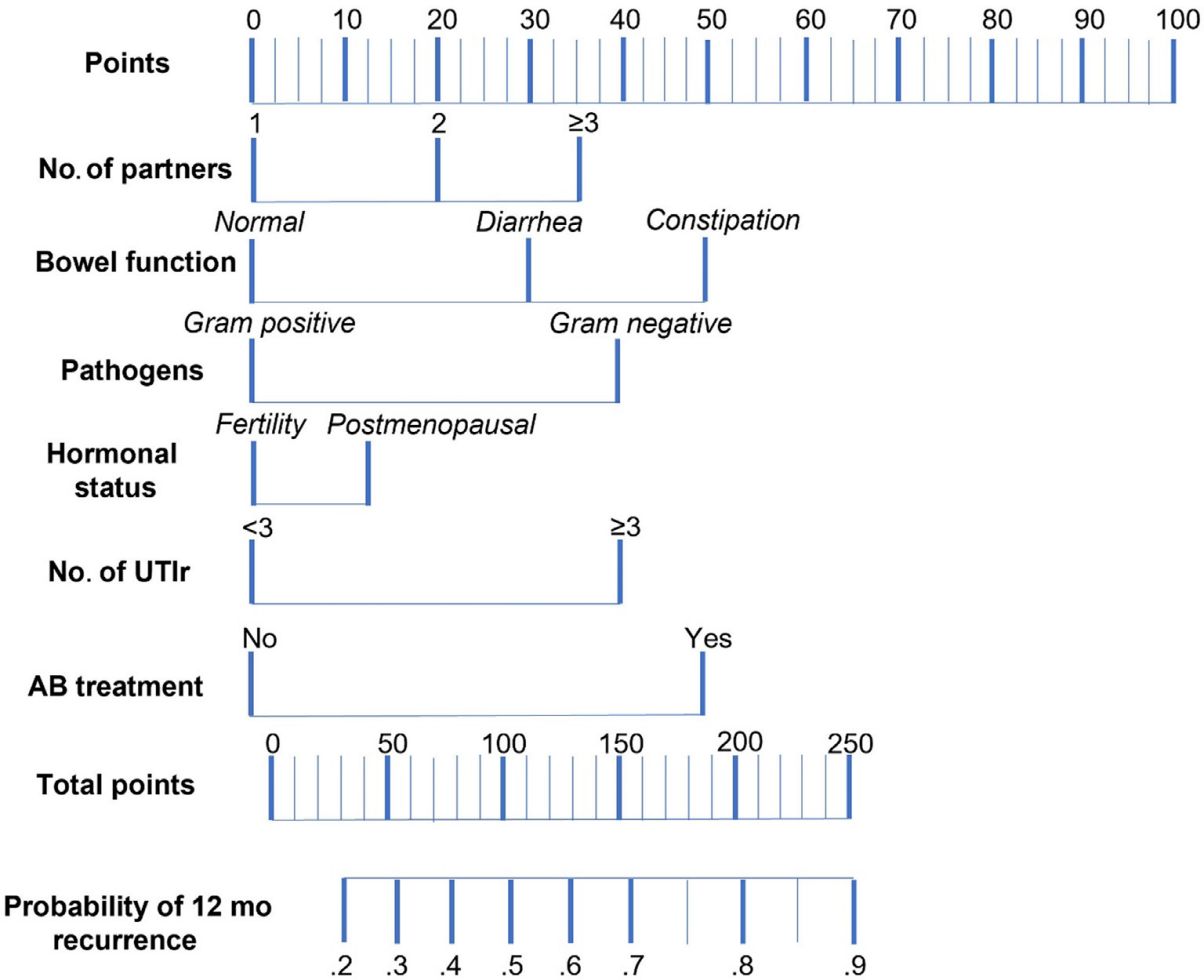
Nomogram to predict 12-mo recurrence risk [38]. AB = antibiotic; UTIr = urinary tract infection recurrence.

In the management of UTIs, a prompt diagnosis, risk identification and early treatment are essential to reduce the number of symptomatic episodes, the level of stress and anxiety of the patients, and the number of unnecessary antibiotics. Recently, Cai et al. [2], [38] developed and validated a nomogram that has been proved easy to use and accurate in predicting the recurrence risk in women affected by rUTIs. Nomogram variables included the number of partners, bowel function, type of pathogens isolated (Gram-positive/negative), hormonal status, number of previous UTI recurrences, and previous treatment of asymptomatic bacteriuria (Fig. 1).

**Fig. 2:**
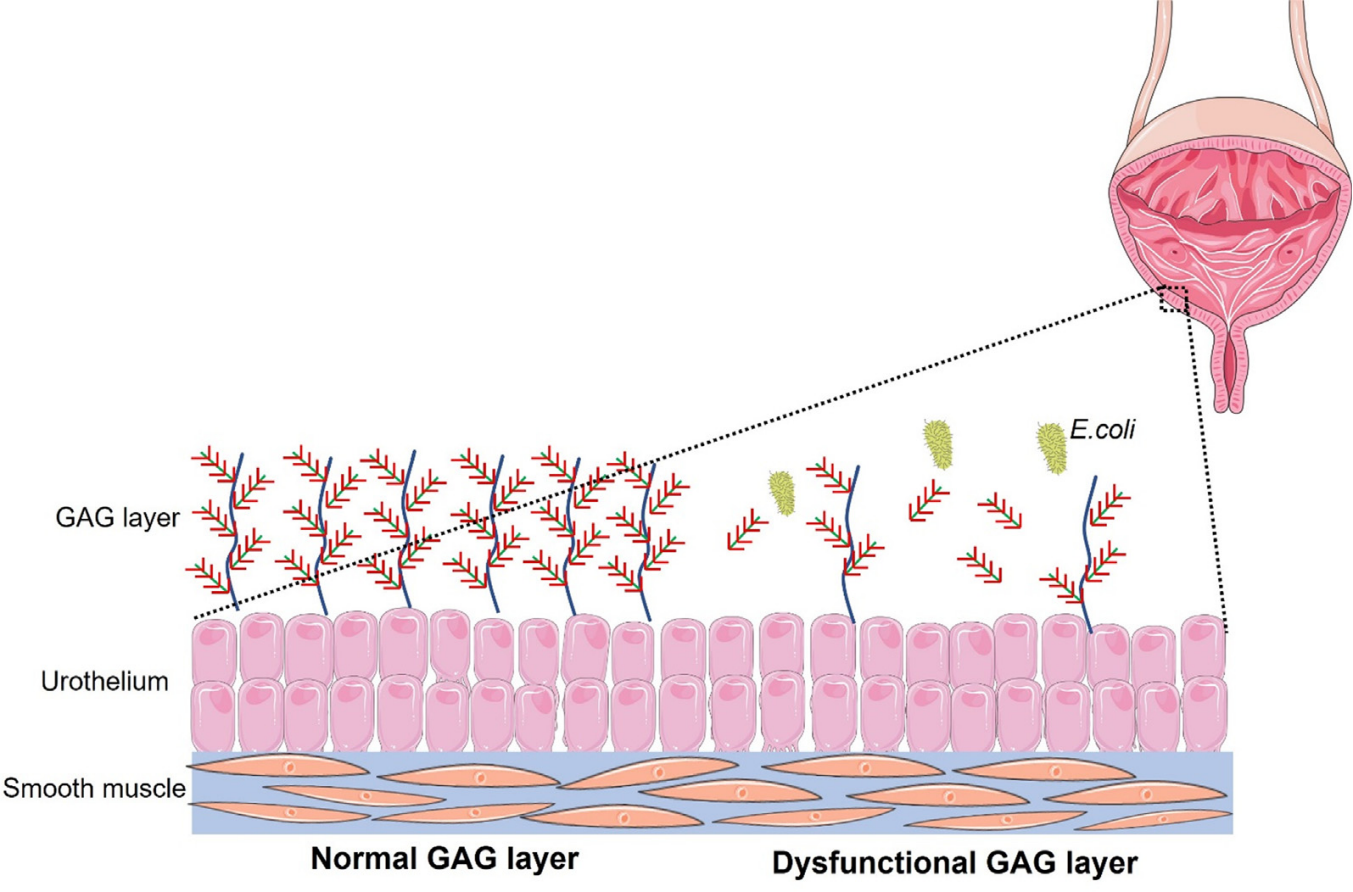
Schematic representation of the GAG layer in a physiological and pathological condition. A dysfunctional GAG layer allows bacteria to penetrate the bladder causing UTIs. GAG = glycosaminoglycan; UTI = urinary tract infection.

Considering the evident limits of the antibiotics for the prevention of episodes of rUTIs, alternative treatments focus on the relationship between bacteria and host. Bladder epithelium is a specialized tissue protected by GAGs, such as HA and CS [39]. The GAG layer is a protective barrier, preventing damaging substances found in the urine from penetrating into the deeper layers of the bladder wall [40]. The presence of an intact GAG layer covering the urothelium prevents bacteria (especially *E. coli*) from penetrating the bladder, and attacking and destroying urothelial cells (Fig. 2).

A deficit in the GAG layer is the first step in developing chronic inflammatory diseases of the bladder [41]. As a matter of fact, in chronic inflammatory bladder diseases and different types of cystitis, there is a deficit of GAGs in the bladder lining [42], [43]. Damage of the GAG layer leads to the loss of the “watertight” function and allows both normal ions (ie, H^+^, K^+^, Na^+^, and Cl^–^) and abnormal constituents of urine (ie, metabolites of cytotoxic drugs or toxic substances excreted into it) to come into direct contact with the subepithelial layers [41].

Intravesical formulations of HA and CS, alone or in combination and at different concentrations, aim to re-establish epithelial integrity by binding to proteoglycans or interacting with structural urothelium.

Exogenous GAGs, especially in the combination of HA + CS and CaCl_2_ (Ialuril; IBSA, Lugano, Switzerland), were originally investigated for efficacy in patients with painful bladder syndrome and interstitial cystitis who had not benefited from other therapies [44]. More recent studies showed its efficacy in preventing rUTIs in patients with at least three episodes of bacterial cystitis in the previous year [45]. Early observational studies suggested that exogenous intravesical HA could reduce the frequency of episodes of rUTIs [46], [47]. Subsequent prospective studies investigated the combination of HA + CS + CaCl_2_ in patients with rUTIs [45]. A randomized, double-blind, placebo-controlled trial of HA + CS + CaCl_2_ (four instillations at weekly intervals, then five instillations at monthly intervals) monitored for 12 mo, 28 patients were randomized to HA + CS + CaCl_2_ and 29 patients were randomized to placebo. Patients treated with HA + CS + CaCl_2_ had fewer rUTI episodes, a longer interval free from recurrence, and a greater improvement in the QoL, measured with the SF-36 questionnaire, than patients in the placebo group (87% vs 10% and 185 vs 53 d; *p* < 0.05 for both); the total number of rUTI episodes experienced at 6 and 12 mo was also significantly lower (*p* < 0.05) in the HA + CS + CaCl_2_ group. Symptoms (pelvic pain, urgency, and frequency score) improved significantly, and QoL improved as well through 12 mo (*p* < 0.001) [45]. De Vita and Giordano [39] compared exogenous intravesical GAGs with antibiotic prophylaxis for rUTIs in 28 women; the intravesical treatment significantly reduced the recurrence of UTIs and improved urinary symptoms, QoL, assessed with the King's Health Questionnaire, and cystometric capacity at 12-mo follow-up. The tolerability of the HA + CS formulation was good, without any serious adverse events reported. Recently, the effectiveness of intravesical instillation of HA + CS + CaCl_2_ as a nonantibiotic treatment option for the prophylaxis of rUTIs in female patients was assessed at seven European centers [48]. Cicione et al. [48] administered 50 ml of a GAG solution to 157 women with rUTIs via intravesical instillation. At 12 mo, the number of UTI episodes decreased, with milder symptoms and improved QoL, measured with the SF-36 questionnaire. Another study compared, from 2009 to 2013, the intravesical administration of HA + CS + CaCl_2_ with the standard of care (antimicrobial/immunoactive prophylaxis/probiotics/cranberry) in 276 women treated for rUTIs. Bladder instillations of the combination of HA + CS + CaCl_2_ reduced the risk of rUTIs compared with the current standard management [49]. The treatment with HA + CS showed clinical benefit up to 36 mo after treatment [50]. Finally, Costantini et al. [51] investigated the endoscopic morphology of bladder mucosa in patients who received intravesical instillation with HA + CS to treat rUTIs, demonstrating significant improvements. The available scientific evidence supports the efficacy and safety of HA + CS in the management of rUTIs. It is known that rUTIs are, for the vast majority of cases, the recurrence of infection caused by *E. coli* [52], [53], while there are no data on the effectiveness of GAG therapy in the management of rUTIs caused by different bacterial species. The therapy improves patients’ QoL and decreases the number of episodes of UTIs, allowing a more rational use of antibiotics. Restoration of the GAG bladder layer seems to be a promising nonantibiotic therapy to prevent rUTIs. Importantly, the safety profile of this combination has been reported to be very favorable, without adverse events of particular significance.

## 4 Conclusions

This paper summarizes the EAU recommendations for the treatment of UTIs/rUTIs, presents data on the adherence to the recommendations, and discusses the evidence on the use of HA + CS in the prophylaxis of rUTIs.

The main limits of this paper are that it is focused on the European approach, and that the survey and its statistical analysis are still preliminary.

The strengths of this paper are that the issues are discussed from different perspectives, as the work is the result of a multinational and multidisciplinary panel of authors. In addition, the survey and the symposium give valuable insight into the attitude of physicians treating UTIs. As a result of the abovementioned considerations, the authors, as far as clinical practice is concerned, recommend performing urine culture for each symptomatic rUTI to investigate the type of bacteria affecting the patient’s bladder. Different bacterial species may be the cause of subsequent episodes of UTIs in the same patient. Moreover, several pathologies can show an overlap in the type of bacteria involved.

The survey showed that amoxicillin, fosfomycin, and nitrofurantoin, alone or in combination, are the most prescribed antibiotics. The authors, adding recent information, report that, in the country most represented in the survey (Italy), there are two scenarios: clinicians prescribing fluoroquinolones alone or fluoroquinolones in combination with cephalosporins or GAGs in the relapses. One point of reflection is the weight that economic or legal concerns may have in the choice of physicians to prescribe antibiotics despite the awareness of the resistance issue.

The authors, commenting on the available data, observed that GAG therapy is an effective alternative treatment to antibiotics. About the intravesical administration of GAGs, the authors suggest one instillation per week, two instillations per month, and one instillation per month until the patient’s recovery. With this approach, the authors obtained good adherence to the schedule and improved QoL of the patients. Table 4 summarizes the considerations and recommendations emerged during the symposium.

**Table 4:** Clinical remarks emerged from the symposium

Background	• UTIs are very common; many women suffer from rUTIs • Evidence shows that antibiotics, estrogen, and immunoactive prophylaxis can reduce the frequency of recurrences • Other preventive measures are not yet evidenced based • Patience and time are needed by patients and physicians • Multimodal therapy leads to success in many cases
Real-world prescribing patterns	• Of the responders, >40% reported that they start antibiotic therapy for the treatment of an rUTI episode as soon as possible (without waiting for antibiogram/culture results) • Changing antibiotic therapy during the treatment of an rUTI episode is frequent • Antibiotic resistance is considered a relevant problem (extremely relevant 40%) • Of the urologists involved in the survey, 60% prescribe antibiotics for the prophylaxis of rUTIs • Of the urologists involved in the survey, >70% prescribe nonantibiotic methods for the prophylaxis of rUTIs and declare that they will continue or start to prescribe nonantibiotic treatment • Intravesical instillation of HA alone or in combination with CS and probiotics is the nonantibiotic treatment considered most effective by the responders
GAG therapy	• Safe and useful in the management of patients with recurrent UTIs • Able to improve patient’s QoL • Decrease in the number of UTI recurrences • Improve the adherence to antimicrobial stewardship

CS = chondroitin sulfate; GAG = glycosaminoglycan; HA = hyaluronic acid; QoL = quality of life; rUTI = recurrent UTI; UTI = urinary tract infection.

In conclusion, adherence to the international guidelines is important to align the clinical practice and avoid the spreading of antibiotic resistance. The survey outlines that the misuse and overuse of antibiotics are major problems; the clinical evidence confirms that GAG therapy is a valuable approach to prevent the recurrence of the episodes of UTIs and to limit the onset of antibiotic resistance.

  ***Author contributions*:** Franck Bruyere had full access to all the data in the study and takes responsibility for the integrity of the data and the accuracy of the data analysis.

*Study concept and design*: Bonkat, Cai, Galeone, Koves, Bruyere.

*Acquisition of data*: Bonkat, Cai, Galeone, Koves, Bruyere.

*Analysis and interpretation of data*: Bonkat, Cai, Galeone, Koves, Bruyere.

*Drafting of the manuscript*: Bonkat, Cai, Galeone, Koves.

*Critical revision of the manuscript for important intellectual content*: Bonkat, Cai, Galeone, Koves, Bruyere.

*Statistical analysis*: None.

*Obtaining funding*: Bonkat, Cai, Galeone, Koves.

*Administrative, technical, or material support*: Bonkat, Cai, Galeone, Koves.

*Supervision*: Bonkat, Cai, Galeone, Koves, Bruyere.

*Other*: None.

  ***Financial disclosures:*** Franck Bruyere certifies that all conflicts of interest, including specific financial interests and relationships and affiliations relevant to the subject matter or materials discussed in the manuscript (eg, employment/affiliation, grants or funding, consultancies, honoraria, stock ownership or options, expert testimony, royalties, or patents filed, received, or pending), are the following: Financial interests: Gernot Bonkat, Tommaso Cai, Carlotta Galeone, Bela Koves, and Franck Bruyere have received speaker honorarium from IBSA. Nonfinancial interests: none.

  ***Funding/Support and role of the sponsor*:** The publication of this article was supported by a nonconditioning grant from IBSA.

  ***Acknowledgments*:** Dr. Salvatore Bianco of AKROS BioScience (Pomezia, Italy) provided medical writing and editing services during the preparation of the paper.

## References

[1] Bono M.J., Reygaert W.C. (2021).

[2] Cai T. (2021). Recurrent uncomplicated urinary tract infections: definitions and risk factors. GMS Infect Dis.

[3] Geerlings S.E. (2016). Clinical presentations and epidemiology of urinary tract infections. Microbiol Spectr.

[4] Flores-Mireles A.L., Walker J.N., Caparon M., Hultgren S.J. (2015). Urinary tract infections: epidemiology, mechanisms of infection and treatment options. Nat Rev Microbiol.

[5] Ronald A.R., Nicolle L.E., Stamm E. (2001). Urinary tract infection in adults: research priorities and strategies. Int J Antimicrob Agents.

[6] Epp A., Larochelle A. (2010). Recurrent urinary tract infection. J Obstet Gynaecol Can.

[7] Prevention of recurrent urinary tract infections in women. Drug Ther Bull 2013;51:69–72. 10.1136/dtb.2013.6.0187 23766394

[8] Cai T, Tamanini I, Collini L, et al. Management of recurrent cystitis in women: when prompt identification of risk factors might make a difference. Eur Urol Focus. In press. 10.1016/j.euf.2022.01.014. 35135727

[9] Sihra N., Goodman A., Zakri R., Sahai A., Malde S. (2018). Nonantibiotic prevention and management of recurrent urinary tract infection. Nat Rev Urol.

[10] Issakhanian L., Behzadi P. (2019). Antimicrobial agents and urinary tract infections. Curr Pharm Des.

[11] Wagenlehner F.M., Vahlensieck W., Bauer H.W., Weidner W., Piechota H.J., Naber K.G. (2013). Prevention of recurrent urinary tract infections. Minerva Urol Nefrol.

[12] Lorenzo-Gómez M.F., Padilla-Fernández B., Flores-Fraile J. (2021). Impact of whole-cell bacterial immunoprophylaxis in the management of recurrent urinary tract infections in the frail elderly. Vaccine.

[13] Naber K.G., Cho Y.H., Matsumoto T., Schaeffer A.J. (2009). Immunoactive prophylaxis of recurrent urinary tract infections: a meta-analysis. Int J Antimicrob Agents.

[14] Rozenberg S., Pastijn A., Gevers R., Murillo D. (2004). Estrogen therapy in older patients with recurrent urinary tract infections: a review. Int J Fertil Womens Med.

[15] Stothers L. (2009). Should hormone replacement therapy be used in postmenopausal women for voiding dysfunction?. Can Urol Assoc J.

[16] EAU guidelines. Presented at the EAU Annual Congress, Milan, Italy. Arnhem, the Netherlands: EAU Guidelines Office; 2021. http://uroweb.org/guidelines/compilations-of-all-guidelines/.

[17] EAU guidelines on urological infections. Presented at the EAU Annual Congress, Amsterdam, the Netherlands; 2022. Arnhem, the Netherlands: EAU Guidelines Office; 2022. https://uroweb.org/guidelines/urological-infections/summary-of-changes.

[18] Bader M.S., Loeb M., Leto D., Brooks A.A. (2020). Treatment of urinary tract infections in the era of antimicrobial resistance and new antimicrobial agents. Postgrad Med.

[19] Bader M.S., Loeb M., Brooks A.A. (2017). An update on the management of urinary tract infections in the era of antimicrobial resistance. Postgrad Med.

[20] Aslam B., Wang W., Arshad M.I. (2018). Antibiotic resistance: a rundown of a global crisis. Infect Drug Resist.

[21] Price R. (2016). O'Neill report on antimicrobial resistance: funding for antimicrobial specialists should be improved. Eur J Hosp Pharm.

[22] Laxminarayan R., Duse A., Wattal C. (2013). Antibiotic resistance-the need for global solutions. Lancet Infect Dis.

[23] Jonas O.B., Irwin A., Berthe F.C.J., Le Gall F.G., Marquez P.V. (2017).

[24] Shaskolskiy B., Dementieva E., Leinsoo A. (2016). Drug resistance mechanisms in bacteria causing sexually transmitted diseases and associated with vaginosis. Front Microbiol.

[25] Cai T., Tamanini I., Kulchavenya E. (2017). The role of nutraceuticals and phytotherapy in the management of urinary tract infections: what we need to know?. Arch Ital Urol Androl.

[26] Andersson M.I., MacGowan A.P. (2003). Development of the quinolones. J Antimicrob Chemother.

[27] Bonkat G., Wagenlehner F. (2019). In the line of fire: should urologists stop prescribing fluoroquinolones as default?. Eur Urol.

[28] Bonkat G., Pilatz A., Wagenlehner F. (2019). Time to adapt our practice? The European Commission has restricted the use of fluoroquinolones since March 2019. Eur Urol.

[29] Cai T., Verze P., Brugnolli A. (2016). Adherence to European Association of Urology guidelines on prophylactic antibiotics: an important step in antimicrobial stewardship. Eur Urol.

[30] Çek M., Tandoğdu Z., Naber K. (2013). Antibiotic prophylaxis in urology departments, 2005–2010. Eur Urol.

[31] Bausch K., Roth J.A., Seifert H.H., Widmer A.F. (2018). Overuse of antimicrobial prophylaxis in low-risk patients undergoing transurethral resection of the prostate. Swiss Med Wkly.

[32] Durkin M.J., Keller M., Butler A.M. (2018). An assessment of inappropriate antibiotic use and guideline adherence for uncomplicated urinary tract infections. Open Forum Infect Dis.

[33] Naber K.G., Bonkat G., Wagenlehner F.M.E. (2020). The EAU and AUA/CUA/SUFU guidelines on recurrent urinary tract infections: what is the difference?. Eur Urol.

[34] Takhar S.S., Moran G.J. (2014). Diagnosis and management of urinary tract infection in the emergency department and outpatient settings. Infect Dis Clin North Am.

[35] Zhu H., Chen Y., Hang Y. (2021). Impact of inappropriate empirical antibiotic treatment on clinical outcomes of urinary tract infections caused by *Escherichia coli*: a retrospective cohort study. J Glob Antimicrob Resist.

[36] Rodriguez-Gómez J., Pérez-Nadales E., Gutiérrez-Gutiérrez B. (2019). Prognosis of urinary tract infection caused by KPC-producing *Klebsiella pneumoniae*: the impact of inappropriate empirical treatment. J Infect.

[37] Robinson T.F., Barsoumian A.E., Aden J.K., Giancola S.E. (2020). Evaluation of the trends and appropriateness of fluoroquinolone use in the outpatient treatment of acute uncomplicated cystitis at five family practice clinics. J Clin Pharm Ther.

[38] Cai T., Mazzoli S., Migno S. (2014). Development and validation of a nomogram predicting recurrence risk in women with symptomatic urinary tract infection. Int J Urol.

[39] De Vita D., Giordano S. (2012). Effectiveness of intravesical hyaluronic acid/chondroitin sulfate in recurrent bacterial cystitis: a randomized study. Int Urogynecol J.

[40] Min G., Zhou G., Schapira M., Sun T.T., Kong X.P. (2003). Structural basis of urothelial permeability barrier function as revealed by Cryo-EM studies of the 16 nm uroplakin particle. J Cell Sci.

[41] Lazzeri M., Hurle R., Casale P., Buffi N., Lughezzani G., Fiorini G. (2016). Managing chronic bladder diseases with the administration of exogenous glycosaminoglycans: an update on the evidence. Ther Adv Urol.

[42] Stellavato A., Pirozzi A.V.A., Diana P. (2019). Hyaluronic acid and chondroitin sulfate, alone or in combination, efficiently counteract induced bladder cell damage and inflammation. PLoS One.

[43] Cervigni M., Natale F., Nasta L., Mako A. (2012). Intravesical hyaluronic acid and chondroitin sulphate for bladder pain syndrome/interstitial cystitis: long-term treatment results. Int Urogynecol J.

[44] Cervigni M., Sommariva M., Tenaglia R. (2017). A randomized, open-label, multicenter study of the efficacy and safety of intravesical hyaluronic acid and chondroitin sulfate versus dimethyl sulfoxide in women with bladder pain syndrome/interstitial cystitis. Neurourol Urodyn.

[45] Damiano R., Quarto G., Bava I. (2011). Prevention of recurrent urinary tract infections by intravesical administration of hyaluronic acid and chondroitin sulphate: a placebo-controlled randomised trial. Eur Urol.

[46] Constantinides C., Manousakas T., Nikolopoulos P., Stanitsas A., Haritopoulos K., Giannopoulos A. (2004). Prevention of recurrent bacterial cystitis by intravesical administration of hyaluronic acid: a pilot study. BJU Int.

[47] Lipovac M., Kurz C., Reithmayr F., Verhoeven H.C., Huber J.C., Imhof M. (2007). Prevention of recurrent bacterial urinary tract infections by intravesical instillation of hyaluronic acid. Int J Gynaecol Obstet.

[48] Cicione A., Cantiello F., Ucciero G. (2014). Intravesical treatment with highly-concentrated hyaluronic acid and chondroitin sulphate in patients with recurrent urinary tract infections: results from a multicentre survey. Can Urol Assoc J.

[49] Ciani O., Arendsen E., Romancik M. (2016). Intravesical administration of combined hyaluronic acid (HA) and chondroitin sulfate (CS) for the treatment of female recurrent urinary tract infections: a European multicentre nested case-control study. BMJ Open.

[50] De Vita D., Madonia M., Coppola E. (2018). Long-term efficacy of intravesical instillation of hyaluronic acid/chondroitin sulfate in recurrent bacterial cystitis: 36 months' follow-up. Clin Exp Obstet Gynecol.

[51] Costantini E., Lazzeri M., Pistolesi D. (2013). Morphological changes of bladder mucosa in patients who underwent instillation with combined sodium hyaluronic acid-chondroitin sulphate (Ialuril®). Urol Int.

[52] Nielsen K.L., Stegger M., Kiil K. (2021). *Escherichia coli* causing recurrent urinary tract infections: comparison to non-recurrent isolates and genomic adaptation in recurrent infections. Microorganisms.

[53] Al-Badr A., Al-Shaikh G. (2013). Recurrent urinary tract infections management in women: a review. Sultan Qaboos Univ Med J.

