# Supplementary material for: Adherence to European Association of Urology Guidelines and State of the Art of Glycosaminoglycan Therapy for the Management of Urinary Tract Infections: A Narrative Review and Expert Meeting Report
Source: Eur Urol Open Sci. 2022 Aug 23;44:37–45. doi: 10.1016/j.euros.2022.07.009 (PMC9424561; doi:10.1016/j.euros.2022.07.009)
Supplement: Supplementary data 1 [file mmc1.docx]

**Supplementary Table 1. Complete list of Countries of origin of the respondents to the survey**

| **Country** | **Percentage of respondents** |
| --- | --- |
| Italy | 26.4% |
| Egypt | 5.1% |
| United Kingdom | 4.6% |
| Denmark | 3.7% |
| Algery | 3.7% |
| Bulgaria | 3.2% |
| Spain | 3.2% |
| Slovenia | 3.2% |
| Serbia | 2.3% |
| Lithuania | 2.3% |
| Austria | 2.3% |
| Slovakia | 2.3% |
| Belorus | 1.9% |
| Polland | 1.9% |
| Romania | 1.9% |
| France | 1.9% |
| Hungary | 1.4% |
| Ukrain | 1.4% |
| Russia | 1.4% |
| Morocco | 1.4% |
| Greece | 0.9% |
| Belgium | 0.9% |
| Finland | 0.9% |
| Portugal | 0.9% |
| Cezch Republic | 0.9% |
| Sweden | 0.9% |
| Netherlands | 0.9% |
| Jordan | 0.9% |
| Iran | 0.9% |
| Brazil | 0.9% |
| Albania | 0.9% |
| Norwey | 0.9% |
| Argentina | 0.9% |
| Kosovo | 0.9% |
| Pakistan | 0.9% |
| Lebanon | 0.9% |
| South Korea | 0.9% |
| Turkey | 0.9% |
| Germany | 0.5% |
| Tunisia | 0.5% |
| Mexico | 0.5% |
| Taiwan | 0.5% |
| India | 0.5% |
| Switzerland | 0.5% |
| Colombia | 0.5% |
| Palestine | 0.5% |
| Oman | 0.5% |
| Uzbekistan | 0.5% |
| United Arab Emirates | 0.5% |
| Kenya | 0.5% |
| Israel | 0.5% |
| Finland | 0.5% |
| Bahrein | 0.5% |
| Japan | 0.5% |
| Armenia | 0.5% |
